# THE EFFECT OF SECONDARY JOBHOLDING ON INCOME MOBILITY OF DIRECT CARE WORKERS

**DOI:** 10.1093/geroni/igae098.0392

**Published:** 2024-12-31

**Authors:** Jiyeon Kim

**Affiliations:** PHI, New York, New York, United States

## Abstract

Recent studies show that many direct care workers acquire secondary jobs because their primary employment does not offer sufficient wages or work hours. While prior research has emphasized how secondary jobs protect direct care workers from poverty, it is unclear how these jobs affect these workers’ subsequent employment outcomes. This paper analyzes longitudinal data to investigate whether secondary jobs can serve as a pathway for direct care workers to obtain a primary job that provides income above the poverty-line and test if some secondary occupations are more beneficial than others. I find that whereas a secondary job in a non-direct care occupation helps direct care workers later attain a primary job that pays above poverty-level, working in two direct care jobs restrains them from doing so. In addition, these effects vary by race and gender such that the benefits to holding a non-direct care secondary job are greater among men and whites, compared to their women and racial minority counterparts. These findings raise concerns about unequal opportunities for wage mobility among direct care workers and the implications this has for the retention of this workforce.

